# {2,2′-[6,6′-Dimethoxy­cyclo­hexane-1,2-diylbis(nitrilo­methyl­idyne)]diphenolato-κ^4^ *O* ^1^,*N*,*N*′,*O* ^1′^}cobalt(II) monohydrate

**DOI:** 10.1107/S1600536809021540

**Published:** 2009-06-13

**Authors:** Yan Bao, Hong-Feng Li, Peng-Fei Yan, Guang-Ming Li, Guang-Feng Hou

**Affiliations:** a Key Laboratory of Functional Inorganic Material Chemistry (Heilongjiang University), Ministry of Education, School of Chemistry and Materials Science, Heilongjiang University, Harbin 150080, People’s Republic of China

## Abstract

In the title complex, [Co(C_22_H_24_N_2_O_4_)]·H_2_O, the Co^II^ atom is in an almost square-planar coordination environment involv­ing two O and two N atoms from the Schiff base ligand. A water mol­ecule cocrystallizes with the coordination compound and may be held in the crystal by O—H⋯O hydrogen bonds. Heteroatomic π–π ring inter­actions may be present between symmetry-related complexes, with centroid–centroid distances of 3.5661 (8) Å.

## Related literature

For related platinum complexes of a similar Schiff base, see: Lu *et al.* (2008 ▶).
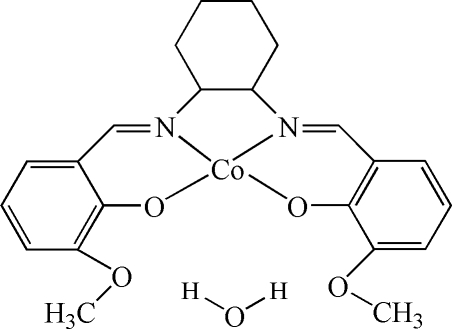


## Experimental

### 

#### Crystal data

[Co(C_22_H_24_N_2_O_4_)]·H_2_O
*M* *_r_* = 457.38
Monoclinic, 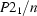

*a* = 11.241 (3) Å
*b* = 10.605 (3) Å
*c* = 17.864 (7) Å
β = 107.158 (14)°
*V* = 2034.9 (12) Å^3^
*Z* = 4
Mo *K*α radiation
μ = 0.88 mm^−1^
*T* = 291 K
0.20 × 0.19 × 0.17 mm

#### Data collection

Rigaku R-AXIS RAPID diffractometer
Absorption correction: multi-scan (*ABSCOR*; Higashi, 1995 ▶) *T*_min_ = 0.826, *T*_max_ = 0.851
19221 measured reflections
4647 independent reflections
3840 reflections with *I* > 2σ(*I*)
*R*_int_ = 0.034

#### Refinement

*R*[*F* ^2^ > 2σ(*F* ^2^)] = 0.029
*wR*(*F* ^2^) = 0.069
*S* = 1.04
4647 reflections
373 parameters
H atoms treated by a mixture of independent and constrained refinement
Δρ_max_ = 0.36 e Å^−3^
Δρ_min_ = −0.24 e Å^−3^

### 

Data collection: *RAPID-AUTO* (Rigaku, 1998 ▶); cell refinement: *RAPID-AUTO*; data reduction: *RAPID-AUTO*; program(s) used to solve structure: *SHELXS97* (Sheldrick, 2008 ▶); program(s) used to refine structure: *SHELXL97* (Sheldrick, 2008 ▶); molecular graphics: *SHELXTL* (Sheldrick, 2008 ▶); software used to prepare material for publication: *SHELXL97*.

## Supplementary Material

Crystal structure: contains datablocks global, I. DOI: 10.1107/S1600536809021540/cs2106sup1.cif

Structure factors: contains datablocks I. DOI: 10.1107/S1600536809021540/cs2106Isup2.hkl

Additional supplementary materials:  crystallographic information; 3D view; checkCIF report

## Figures and Tables

**Table 1:** Hydrogen-bond geometry (Å, °)

*D*—H⋯*A*	*D*—H	H⋯*A*	*D*⋯*A*	*D*—H⋯*A*
O5—H25⋯O2	0.90 (4)	2.11 (4)	2.916 (3)	149 (3)
O5—H25⋯O1	0.90 (4)	2.45 (4)	3.103 (2)	129 (3)
O5—H26⋯O4	0.89 (4)	2.05 (4)	2.895 (3)	159 (4)
O5—H26⋯O3	0.89 (4)	2.59 (4)	3.248 (2)	131 (3)

**Fig. 1.**
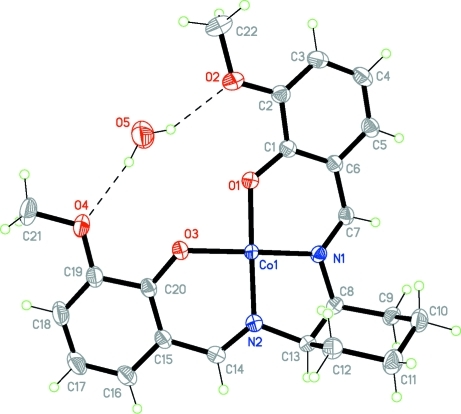
The molecular structure of the title complex, showing 30% probability displacement ellipsoids for non-H atoms. Dashed lines indicate the hydrogen-bonding interactions between the water and the host.

**Fig. 2.**
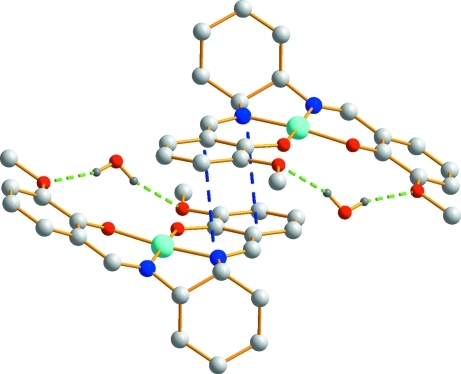
Heteroatomic π–π ring interactions across an inversion center, indicated by broken lines.

## supplementary crystallographic information

### Comment

As shown in Fig. 1, Co^II^ is four-coordinated in a square planar environment as the ligating Schiff base is a tetradentate ligand (Table 1). The co-crystallized water molecule does not coordinate to the Co ion. Its position is stabilized by bifurcated O—H···O hydrogen bonds (Table 2). It is also worth noting that stabilizing π–π ring interactions may occur between symmetry center related phenyl C1 -> C6 and C7 -> O1 heteroatomic rings, as show in Fig. 2. The π–π center to center distance is 3.5661 (8) Å. Together with an almost perfect rings-to-rings matching this may indicate appreciable interactions.

### Experimental

The title complex was obtained by the treatment of cobalt(II) acetate tetrahydrate with the neutral Schiff base in methanol/acetone (1:2). The yellow clear mixture turned to salmon pink precipitation after stirred for 4 h; diethyl ether was allowed to diffuse slowly into the solution of the filtrate. Red single crystals were obtained after several days.Analysis calculated for C_22_H_26_Co_N_2_O_5: C, 57.77; H, 5.73; N, 6.12; Co, 12.88; found: C, 57.56; H, 5.23; N, 6.77; Co, 12.79%.

### Refinement

All H atoms were located in difference Fourier maps and freely refined, but water H atoms was set *U*_iso_(H) = 1.5*U*_eq_(O).

### Crystal data

**Table:**

[Co(C_22_H_24_N_2_O_4_)]·H_2_O	*F*(000) = 956
*M**_r_* = 457.38	*D*_x_ = 1.493 Mg m^−^^3^
Monoclinic, *P*2_1_/*n*	Mo *K*α radiation, λ = 0.71073 Å
Hall symbol: -P 2yn	Cell parameters from 15295 reflections
*a* = 11.241 (3) Å	θ = 3.1–27.5°
*b* = 10.605 (3) Å	µ = 0.88 mm^−^^1^
*c* = 17.864 (7) Å	*T* = 291 K
β = 107.158 (14)°	Block, red
*V* = 2034.9 (12) Å^3^	0.20 × 0.19 × 0.17 mm
*Z* = 4	

### Data collection

**Table:**

Rigaku R-AXIS RAPID diffractometer	4647 independent reflections
Radiation source: fine-focus sealed tube	3840 reflections with *I* > 2σ(*I*)
graphite	*R*_int_ = 0.034
ω scans	θ_max_ = 27.5°, θ_min_ = 3.1°
Absorption correction: multi-scan (*ABSCOR*; Higashi, 1995)	*h* = −14→14
*T*_min_ = 0.826, *T*_max_ = 0.851	*k* = −11→13
19221 measured reflections	*l* = −23→23

### Refinement

**Table:**

Refinement on *F*^2^	Primary atom site location: structure-invariant direct methods
Least-squares matrix: full	Secondary atom site location: difference Fourier map
*R*[*F*^2^ > 2σ(*F*^2^)] = 0.029	Hydrogen site location: inferred from neighbouring sites
*wR*(*F*^2^) = 0.069	H atoms treated by a mixture of independent and constrained refinement
*S* = 1.04	*w* = 1/[σ^2^(*F*_o_^2^) + (0.0317*P*)^2^ + 0.4*P*] where *P* = (*F*_o_^2^ + 2*F*_c_^2^)/3
4647 reflections	(Δ/σ)_max_ = 0.001
373 parameters	Δρ_max_ = 0.36 e Å^−^^3^
0 restraints	Δρ_min_ = −0.24 e Å^−^^3^

### Fractional atomic coordinates and isotropic or equivalent isotropic displacement parameters (Å^2^)

**Table:**

	*x*	*y*	*z*	*U*_iso_*/*U*_eq_	
C1	0.55207 (14)	0.17711 (15)	0.02619 (9)	0.0336 (3)	
C2	0.47583 (15)	0.27964 (16)	−0.01173 (10)	0.0397 (4)	
C3	0.46233 (17)	0.30829 (18)	−0.08877 (11)	0.0450 (4)	
C4	0.52295 (17)	0.23721 (19)	−0.13239 (11)	0.0476 (4)	
C5	0.59830 (16)	0.13964 (19)	−0.09779 (10)	0.0427 (4)	
C6	0.61536 (14)	0.10945 (15)	−0.01824 (9)	0.0336 (3)	
C7	0.69932 (14)	0.00941 (16)	0.01550 (10)	0.0346 (3)	
C8	0.81705 (14)	−0.13254 (16)	0.11525 (10)	0.0349 (3)	
C9	0.91820 (16)	−0.1510 (2)	0.07467 (11)	0.0448 (4)	
C10	1.01669 (18)	−0.0482 (2)	0.09542 (13)	0.0548 (5)	
C11	1.07198 (18)	−0.0338 (3)	0.18328 (13)	0.0588 (6)	
C12	0.97012 (17)	−0.0087 (2)	0.22218 (12)	0.0462 (4)	
C13	0.87250 (15)	−0.11292 (17)	0.20283 (10)	0.0364 (4)	
C14	0.75527 (16)	−0.12574 (16)	0.29443 (10)	0.0385 (4)	
C15	0.65495 (15)	−0.09883 (17)	0.32630 (10)	0.0386 (4)	
C16	0.6452 (2)	−0.1711 (2)	0.39057 (12)	0.0509 (5)	
C17	0.5519 (2)	−0.1499 (2)	0.42283 (13)	0.0629 (6)	
C18	0.4652 (2)	−0.0550 (2)	0.39271 (13)	0.0602 (6)	
C19	0.47299 (17)	0.01755 (19)	0.33093 (11)	0.0447 (4)	
C20	0.56884 (15)	−0.00204 (16)	0.29425 (10)	0.0365 (4)	
C21	0.2944 (2)	0.1401 (3)	0.33174 (16)	0.0633 (6)	
C22	0.3317 (2)	0.4392 (2)	0.00145 (17)	0.0610 (6)	
N1	0.73023 (11)	−0.02450 (13)	0.08792 (8)	0.0322 (3)	
N2	0.76520 (12)	−0.07817 (13)	0.23028 (8)	0.0351 (3)	
Co1	0.656300 (17)	0.030194 (19)	0.162807 (12)	0.02751 (7)	
O1	0.55821 (10)	0.15217 (11)	0.09913 (7)	0.0371 (3)	
O2	0.42054 (14)	0.34365 (13)	0.03579 (9)	0.0586 (4)	
O3	0.57104 (11)	0.06922 (11)	0.23491 (7)	0.0400 (3)	
O4	0.39176 (12)	0.11321 (15)	0.29806 (8)	0.0559 (4)	
O5	0.30979 (18)	0.1993 (3)	0.13719 (12)	0.0994 (7)	
H25	0.370 (4)	0.239 (4)	0.122 (2)	0.149*	
H26	0.354 (4)	0.176 (4)	0.185 (2)	0.149*	
H5	0.7650 (15)	−0.2112 (17)	0.1078 (10)	0.034 (4)*	
H4	0.7370 (17)	−0.0353 (16)	−0.0216 (11)	0.042 (5)*	
H15	0.8198 (16)	−0.1865 (17)	0.3229 (10)	0.038 (5)*	
H14	0.9086 (16)	−0.1919 (17)	0.2269 (11)	0.039 (5)*	
H6	0.8798 (17)	−0.1612 (17)	0.0162 (12)	0.047 (5)*	
H1	0.4088 (17)	0.3782 (18)	−0.1147 (11)	0.047 (5)*	
H13	0.9278 (18)	0.077 (2)	0.2029 (12)	0.053 (6)*	
H12	1.0042 (19)	−0.0036 (18)	0.2804 (13)	0.051 (6)*	
H2	0.5101 (18)	0.2573 (19)	−0.1860 (13)	0.057 (6)*	
H23	0.261 (2)	0.402 (2)	−0.0407 (14)	0.062 (6)*	
H16	0.704 (2)	−0.237 (2)	0.4099 (13)	0.060 (6)*	
H3	0.6401 (19)	0.087 (2)	−0.1280 (12)	0.058 (6)*	
H7	0.9564 (18)	−0.235 (2)	0.0946 (12)	0.054 (6)*	
H9	0.978 (2)	0.038 (2)	0.0714 (14)	0.065 (7)*	
H19	0.329 (2)	0.168 (2)	0.3868 (16)	0.077 (7)*	
H18	0.396 (2)	−0.039 (2)	0.4151 (15)	0.079 (8)*	
H11	1.132 (2)	0.044 (2)	0.1943 (14)	0.071 (7)*	
H17	0.541 (2)	−0.207 (2)	0.4636 (15)	0.078 (7)*	
H20	0.247 (2)	0.060 (2)	0.3337 (14)	0.068 (7)*	
H22	0.301 (2)	0.471 (2)	0.0463 (17)	0.087 (9)*	
H8	1.082 (2)	−0.065 (2)	0.0701 (13)	0.063 (6)*	
H10	1.116 (2)	−0.111 (2)	0.2077 (14)	0.064 (7)*	
H24	0.368 (2)	0.504 (3)	−0.0252 (16)	0.087 (9)*	
H21	0.245 (2)	0.210 (2)	0.2927 (16)	0.083 (8)*	

### Atomic displacement parameters (Å^2^)

**Table:**

	*U*^11^	*U*^22^	*U*^33^	*U*^12^	*U*^13^	*U*^23^
C1	0.0289 (7)	0.0366 (8)	0.0335 (9)	−0.0053 (6)	0.0065 (6)	0.0020 (7)
C2	0.0366 (8)	0.0387 (9)	0.0430 (10)	−0.0011 (7)	0.0104 (7)	0.0043 (8)
C3	0.0400 (9)	0.0438 (10)	0.0466 (11)	−0.0021 (8)	0.0054 (8)	0.0121 (8)
C4	0.0482 (10)	0.0565 (12)	0.0349 (10)	−0.0083 (9)	0.0072 (8)	0.0100 (9)
C5	0.0422 (9)	0.0514 (11)	0.0341 (9)	−0.0054 (8)	0.0105 (8)	−0.0002 (8)
C6	0.0292 (7)	0.0386 (9)	0.0319 (8)	−0.0049 (7)	0.0074 (6)	0.0008 (7)
C7	0.0323 (7)	0.0409 (9)	0.0315 (8)	−0.0029 (7)	0.0110 (7)	−0.0045 (7)
C8	0.0307 (7)	0.0358 (9)	0.0381 (9)	0.0020 (7)	0.0099 (7)	−0.0035 (7)
C9	0.0385 (9)	0.0572 (12)	0.0393 (10)	0.0107 (8)	0.0125 (8)	−0.0032 (9)
C10	0.0384 (9)	0.0813 (16)	0.0489 (12)	−0.0047 (10)	0.0194 (9)	0.0004 (11)
C11	0.0355 (9)	0.0888 (17)	0.0521 (13)	−0.0097 (11)	0.0130 (9)	−0.0079 (12)
C12	0.0381 (9)	0.0593 (12)	0.0386 (10)	−0.0060 (8)	0.0075 (8)	−0.0066 (9)
C13	0.0319 (8)	0.0415 (9)	0.0360 (9)	0.0060 (7)	0.0105 (7)	0.0040 (7)
C14	0.0409 (9)	0.0395 (9)	0.0337 (9)	0.0009 (7)	0.0092 (7)	0.0034 (7)
C15	0.0404 (8)	0.0454 (10)	0.0303 (8)	−0.0045 (8)	0.0111 (7)	−0.0004 (7)
C16	0.0575 (11)	0.0561 (12)	0.0401 (11)	−0.0021 (10)	0.0162 (9)	0.0087 (9)
C17	0.0711 (14)	0.0784 (16)	0.0467 (12)	−0.0038 (12)	0.0292 (11)	0.0157 (11)
C18	0.0566 (12)	0.0860 (17)	0.0480 (12)	−0.0035 (11)	0.0309 (10)	0.0033 (11)
C19	0.0411 (9)	0.0589 (11)	0.0367 (9)	−0.0019 (8)	0.0157 (8)	−0.0045 (8)
C20	0.0385 (8)	0.0438 (9)	0.0280 (8)	−0.0046 (7)	0.0109 (7)	−0.0039 (7)
C21	0.0442 (11)	0.0938 (19)	0.0601 (15)	−0.0018 (13)	0.0281 (11)	−0.0154 (14)
C22	0.0524 (12)	0.0483 (12)	0.0757 (17)	0.0146 (10)	0.0088 (12)	0.0001 (12)
N1	0.0286 (6)	0.0357 (7)	0.0324 (7)	−0.0002 (6)	0.0091 (5)	−0.0018 (6)
N2	0.0345 (7)	0.0380 (7)	0.0335 (7)	0.0013 (6)	0.0112 (6)	0.0013 (6)
Co1	0.02730 (10)	0.03082 (12)	0.02579 (11)	0.00243 (8)	0.00995 (8)	0.00088 (8)
O1	0.0373 (6)	0.0411 (6)	0.0344 (6)	0.0063 (5)	0.0128 (5)	0.0035 (5)
O2	0.0652 (8)	0.0561 (8)	0.0584 (9)	0.0267 (7)	0.0244 (7)	0.0140 (7)
O3	0.0448 (6)	0.0445 (7)	0.0350 (6)	0.0063 (5)	0.0186 (5)	0.0036 (5)
O4	0.0476 (7)	0.0772 (10)	0.0513 (8)	0.0127 (7)	0.0277 (6)	0.0003 (7)
O5	0.0700 (12)	0.161 (2)	0.0736 (13)	0.0206 (13)	0.0315 (10)	0.0263 (14)

### Geometric parameters (Å, °)

**Table:**

C1—O1	1.3112 (19)	C13—N2	1.477 (2)
C1—C6	1.409 (2)	C13—H14	0.974 (18)
C1—C2	1.426 (2)	C14—N2	1.287 (2)
C2—O2	1.371 (2)	C14—C15	1.434 (2)
C2—C3	1.373 (3)	C14—H15	0.991 (18)
C3—C4	1.398 (3)	C15—C20	1.411 (2)
C3—H1	0.980 (19)	C15—C16	1.411 (2)
C4—C5	1.364 (3)	C16—C17	1.356 (3)
C4—H2	0.95 (2)	C16—H16	0.96 (2)
C5—C6	1.413 (2)	C17—C18	1.394 (3)
C5—H3	0.99 (2)	C17—H17	0.98 (2)
C6—C7	1.430 (2)	C18—C19	1.369 (3)
C7—N1	1.288 (2)	C18—H18	0.99 (3)
C7—H4	1.005 (19)	C19—O4	1.375 (2)
C8—N1	1.491 (2)	C19—C20	1.432 (2)
C8—C13	1.518 (2)	C20—O3	1.308 (2)
C8—C9	1.531 (2)	C21—O4	1.425 (2)
C8—H5	1.005 (17)	C21—H19	0.99 (3)
C9—C10	1.519 (3)	C21—H20	1.01 (2)
C9—H6	1.01 (2)	C21—H21	1.06 (3)
C9—H7	1.01 (2)	C22—O2	1.427 (2)
C10—C11	1.516 (3)	C22—H23	1.00 (2)
C10—H9	1.05 (2)	C22—H22	1.02 (3)
C10—H8	0.98 (2)	C22—H24	0.99 (3)
C11—C12	1.528 (3)	N1—Co1	1.8635 (14)
C11—H11	1.04 (2)	N2—Co1	1.8443 (14)
C11—H10	0.99 (2)	Co1—O1	1.8556 (12)
C12—C13	1.524 (3)	Co1—O3	1.8647 (12)
C12—H13	1.04 (2)	O5—H25	0.90 (4)
C12—H12	1.00 (2)	O5—H26	0.89 (4)
			
O1—C1—C6	124.74 (15)	N2—C13—H14	110.1 (10)
O1—C1—C2	118.30 (15)	C8—C13—H14	109.1 (11)
C6—C1—C2	116.96 (15)	C12—C13—H14	110.2 (10)
O2—C2—C3	125.08 (16)	N2—C14—C15	124.09 (16)
O2—C2—C1	113.72 (15)	N2—C14—H15	118.0 (10)
C3—C2—C1	121.20 (16)	C15—C14—H15	117.9 (10)
C2—C3—C4	120.77 (17)	C20—C15—C16	121.09 (16)
C2—C3—H1	120.7 (11)	C20—C15—C14	120.62 (15)
C4—C3—H1	118.5 (11)	C16—C15—C14	118.29 (17)
C5—C4—C3	119.74 (18)	C17—C16—C15	120.7 (2)
C5—C4—H2	121.1 (12)	C17—C16—H16	120.3 (13)
C3—C4—H2	119.2 (12)	C15—C16—H16	118.9 (13)
C4—C5—C6	120.62 (17)	C16—C17—C18	119.7 (2)
C4—C5—H3	121.0 (12)	C16—C17—H17	119.7 (14)
C6—C5—H3	118.3 (12)	C18—C17—H17	120.3 (14)
C1—C6—C5	120.65 (16)	C19—C18—C17	120.92 (18)
C1—C6—C7	121.38 (15)	C19—C18—H18	118.5 (15)
C5—C6—C7	117.96 (15)	C17—C18—H18	120.6 (14)
N1—C7—C6	125.43 (15)	C18—C19—O4	124.51 (17)
N1—C7—H4	119.3 (11)	C18—C19—C20	121.53 (19)
C6—C7—H4	115.2 (11)	O4—C19—C20	113.96 (16)
N1—C8—C13	105.21 (13)	O3—C20—C15	125.06 (14)
N1—C8—C9	116.68 (14)	O3—C20—C19	118.93 (16)
C13—C8—C9	111.66 (14)	C15—C20—C19	116.01 (15)
N1—C8—H5	107.2 (9)	O4—C21—H19	110.8 (14)
C13—C8—H5	107.2 (10)	O4—C21—H20	108.8 (13)
C9—C8—H5	108.5 (9)	H19—C21—H20	105.9 (19)
C10—C9—C8	112.49 (16)	O4—C21—H21	100.6 (14)
C10—C9—H6	112.8 (11)	H19—C21—H21	115 (2)
C8—C9—H6	110.6 (10)	H20—C21—H21	115.9 (19)
C10—C9—H7	110.1 (11)	O2—C22—H23	109.8 (13)
C8—C9—H7	104.1 (11)	O2—C22—H22	104.5 (15)
H6—C9—H7	106.1 (16)	H23—C22—H22	110.4 (19)
C11—C10—C9	111.82 (18)	O2—C22—H24	111.5 (15)
C11—C10—H9	109.3 (13)	H23—C22—H24	105 (2)
C9—C10—H9	110.1 (12)	H22—C22—H24	116 (2)
C11—C10—H8	111.3 (13)	C7—N1—C8	120.01 (13)
C9—C10—H8	110.0 (13)	C7—N1—Co1	126.08 (11)
H9—C10—H8	104.2 (17)	C8—N1—Co1	113.06 (10)
C10—C11—C12	110.80 (17)	C14—N2—C13	119.46 (14)
C10—C11—H11	108.6 (13)	C14—N2—Co1	127.67 (12)
C12—C11—H11	108.0 (13)	C13—N2—Co1	112.85 (11)
C10—C11—H10	111.9 (14)	N2—Co1—O1	174.19 (6)
C12—C11—H10	106.9 (13)	N2—Co1—N1	85.67 (6)
H11—C11—H10	110.6 (18)	O1—Co1—N1	95.00 (6)
C13—C12—C11	110.88 (17)	N2—Co1—O3	93.68 (6)
C13—C12—H13	109.3 (11)	O1—Co1—O3	86.28 (5)
C11—C12—H13	109.5 (11)	N1—Co1—O3	173.66 (5)
C13—C12—H12	107.7 (11)	C1—O1—Co1	126.68 (10)
C11—C12—H12	112.0 (12)	C2—O2—C22	118.21 (17)
H13—C12—H12	107.3 (16)	C20—O3—Co1	124.61 (11)
N2—C13—C8	104.42 (13)	C19—O4—C21	117.60 (18)
N2—C13—C12	110.35 (14)	H25—O5—H26	99 (3)
C8—C13—C12	112.49 (15)		

### Hydrogen-bond geometry (Å, °)

**Table:**

*D*—H···*A*	*D*—H	H···*A*	*D*···*A*	*D*—H···*A*
O5—H25···O2	0.90 (4)	2.11 (4)	2.916 (3)	149 (3)
O5—H25···O1	0.90 (4)	2.45 (4)	3.103 (2)	129 (3)
O5—H26···O4	0.89 (4)	2.05 (4)	2.895 (3)	159 (4)
O5—H26···O3	0.89 (4)	2.59 (4)	3.248 (2)	131 (3)
